# Bis{5-methoxy-2-[(2-morpholinoethyl)iminomethyl-κ*N*]phenolato-κ*O* ^1^}nickel(II)

**DOI:** 10.1107/S1600536809020790

**Published:** 2009-06-06

**Authors:** Nooraziah Mohd Lair, Hapipah Mohd Ali, Seik Weng Ng

**Affiliations:** a Department of Chemistry, University of Malaya, 50603 Kuala Lumpur, Malaysia

## Abstract

The asymmetric unit of the crystal structure of the title compound, [Ni(C_14_H_19_N_2_O_3_)_2_], contains two independent Ni^II^ complex mol­ecules, with the metal atoms each located on a center of inversion. Each metal atom is chelated by two Schiff base anions in a distorted square-planar coordination environment.

## Related literature

The Schiff base exists in the zwitterionic form; see: Mohd Lair *et al.* (2009 ▶).
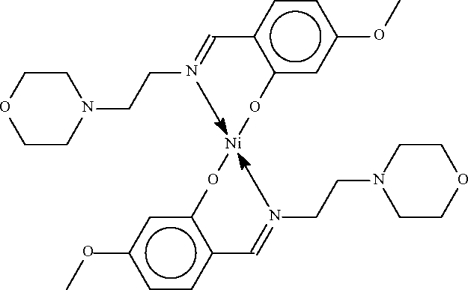


## Experimental

### 

#### Crystal data

[Ni(C_14_H_19_N_2_O_3_)_2_]
*M* *_r_* = 585.33
Triclinic, 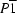

*a* = 10.3358 (1) Å
*b* = 10.4502 (1) Å
*c* = 14.8764 (2) Å
α = 72.482 (1)°
β = 78.847 (1)°
γ = 61.926 (1)°
*V* = 1349.45 (3) Å^3^
*Z* = 2
Mo *K*α radiation
μ = 0.77 mm^−1^
*T* = 100 K
0.45 × 0.35 × 0.25 mm

#### Data collection

Bruker SMART APEX diffractometer
Absorption correction: multi-scan (*SADABS*; Sheldrick, 1996 ▶) *T*_min_ = 0.724, *T*_max_ = 0.831
10299 measured reflections
5978 independent reflections
5388 reflections with *I* > 2σ(*I*)
*R*_int_ = 0.014

#### Refinement

*R*[*F* ^2^ > 2σ(*F* ^2^)] = 0.026
*wR*(*F* ^2^) = 0.079
*S* = 1.02
5978 reflections
357 parameters
H-atom parameters constrained
Δρ_max_ = 0.24 e Å^−3^
Δρ_min_ = −0.45 e Å^−3^

### 

Data collection: *APEX2* (Bruker, 2008 ▶); cell refinement: *SAINT* (Bruker, 2008 ▶); data reduction: *SAINT*; program(s) used to solve structure: *SHELXS97* (Sheldrick, 2008 ▶); program(s) used to refine structure: *SHELXL97* (Sheldrick, 2008 ▶); molecular graphics: *X-SEED* (Barbour, 2001 ▶); software used to prepare material for publication: *publCIF* (Westrip, 2009 ▶).

## Supplementary Material

Crystal structure: contains datablocks global, I. DOI: 10.1107/S1600536809020790/xu2537sup1.cif

Structure factors: contains datablocks I. DOI: 10.1107/S1600536809020790/xu2537Isup2.hkl

Additional supplementary materials:  crystallographic information; 3D view; checkCIF report

## Figures and Tables

**Table 1:** Selected bond lengths (Å)

Ni1—O1	1.9001 (9)
Ni1—N1	2.0077 (10)
Ni2—O4	1.8873 (9)
Ni2—N3	2.0105 (10)

**Fig. 1.**
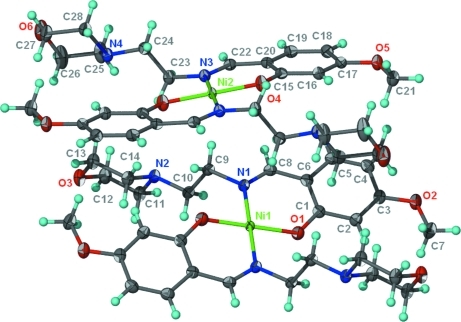
Thermal ellipsoid plot (Barbour, 2001) of the two independent molecules of Ni(C14H19N2O3)2 at the 70% probability level. Hydrogen atoms are drawn as spheres of arbitrary radius.

## supplementary crystallographic information

### Experimental

The Schiff base was synthesized as described (Mohd Lair *et al.*, 2009). The Schiff base (0.25 g, 2 mmol) and nickel(II) acetate (0.29 g, 1 mmol) were heated in ethanol (50 ml) for 5 hours. Large crystals appeared after a day.

### Refinement

Hydrogen atoms were placed at calculated positions (C–H 0.95–0.98 Å) and were treated as riding on their parent carbon atoms, with *U*(H) set to 1.2–1.5 times *U*_eq_(*C*).

### Crystal data

**Table:**

[Ni(C_14_H_19_N_2_O_3_)_2_]	*Z* = 2
*M**_r_* = 585.33	*F*(000) = 620
Triclinic, *P*1	*D*_x_ = 1.441 Mg m^−^^3^
Hall symbol: -P 1	Mo *K*α radiation, λ = 0.71073 Å
*a* = 10.3358 (1) Å	Cell parameters from 7081 reflections
*b* = 10.4502 (1) Å	θ = 2.4–28.3°
*c* = 14.8764 (2) Å	µ = 0.77 mm^−^^1^
α = 72.482 (1)°	*T* = 100 K
β = 78.847 (1)°	Block, brown
γ = 61.926 (1)°	0.45 × 0.35 × 0.25 mm
*V* = 1349.45 (3) Å^3^	

### Data collection

**Table:**

Bruker SMART APEX diffractometer	5978 independent reflections
Radiation source: fine-focus sealed tube	5388 reflections with *I* > 2σ(*I*)
graphite	*R*_int_ = 0.014
ω scans	θ_max_ = 27.5°, θ_min_ = 1.4°
Absorption correction: multi-scan (*SADABS*; Sheldrick, 1996)	*h* = −13→13
*T*_min_ = 0.724, *T*_max_ = 0.831	*k* = −13→13
10299 measured reflections	*l* = −19→18

### Refinement

**Table:**

Refinement on *F*^2^	Primary atom site location: structure-invariant direct methods
Least-squares matrix: full	Secondary atom site location: difference Fourier map
*R*[*F*^2^ > 2σ(*F*^2^)] = 0.026	Hydrogen site location: inferred from neighbouring sites
*wR*(*F*^2^) = 0.079	H-atom parameters constrained
*S* = 1.02	*w* = 1/[σ^2^(*F*_o_^2^) + (0.0486*P*)^2^ + 0.3802*P*] where *P* = (*F*_o_^2^ + 2*F*_c_^2^)/3
5978 reflections	(Δ/σ)_max_ = 0.001
357 parameters	Δρ_max_ = 0.24 e Å^−^^3^
0 restraints	Δρ_min_ = −0.45 e Å^−^^3^

### Fractional atomic coordinates and isotropic or equivalent isotropic displacement parameters (Å^2^)

**Table:**

	*x*	*y*	*z*	*U*_iso_*/*U*_eq_	
Ni1	0.5000	0.5000	0.5000	0.01251 (7)	
Ni2	0.0000	0.5000	0.5000	0.01139 (7)	
O1	0.64797 (11)	0.38684 (10)	0.41957 (7)	0.0219 (2)	
O2	0.85765 (11)	0.09562 (10)	0.19085 (7)	0.0217 (2)	
O3	0.11665 (11)	0.31275 (11)	0.96822 (7)	0.0226 (2)	
O4	0.07976 (11)	0.42908 (10)	0.38957 (6)	0.0201 (2)	
O5	0.33314 (11)	0.09974 (10)	0.18575 (7)	0.0204 (2)	
O6	−0.43863 (13)	0.32859 (12)	0.95338 (8)	0.0333 (3)	
N1	0.40989 (12)	0.35698 (11)	0.53370 (7)	0.0165 (2)	
N2	0.25322 (12)	0.28136 (11)	0.78382 (8)	0.0171 (2)	
N3	0.00294 (12)	0.30250 (11)	0.57283 (7)	0.0160 (2)	
N4	−0.25854 (12)	0.37022 (11)	0.78592 (8)	0.0171 (2)	
C1	0.65173 (14)	0.28594 (14)	0.38318 (9)	0.0169 (2)	
C2	0.75883 (14)	0.24097 (13)	0.30890 (9)	0.0177 (3)	
H2	0.8281	0.2812	0.2890	0.021*	
C3	0.76314 (14)	0.13913 (14)	0.26538 (9)	0.0175 (3)	
C4	0.66694 (15)	0.07116 (14)	0.29655 (9)	0.0199 (3)	
H4	0.6729	−0.0013	0.2677	0.024*	
C5	0.56494 (14)	0.11209 (13)	0.36923 (9)	0.0182 (3)	
H5	0.5003	0.0664	0.3907	0.022*	
C6	0.55269 (14)	0.22006 (13)	0.41341 (9)	0.0163 (2)	
C7	0.94834 (15)	0.17079 (15)	0.15000 (10)	0.0242 (3)	
H7A	1.0063	0.1341	0.0949	0.036*	
H7B	1.0146	0.1511	0.1967	0.036*	
H7C	0.8862	0.2786	0.1304	0.036*	
C8	0.44220 (14)	0.25732 (13)	0.48793 (9)	0.0168 (2)	
H8	0.3858	0.2025	0.5060	0.020*	
C9	0.29494 (14)	0.36405 (14)	0.61125 (9)	0.0175 (2)	
H9A	0.2377	0.3156	0.6020	0.021*	
H9B	0.2268	0.4698	0.6103	0.021*	
C10	0.36353 (14)	0.28583 (14)	0.70611 (9)	0.0179 (3)	
H10A	0.4373	0.1824	0.7049	0.022*	
H10B	0.4148	0.3389	0.7171	0.022*	
C11	0.32412 (15)	0.17738 (14)	0.87014 (9)	0.0202 (3)	
H11A	0.3874	0.2113	0.8890	0.024*	
H11B	0.3868	0.0767	0.8586	0.024*	
C12	0.20802 (15)	0.17037 (14)	0.94837 (9)	0.0219 (3)	
H12A	0.1467	0.1342	0.9296	0.026*	
H12B	0.2564	0.0981	1.0062	0.026*	
C13	0.04749 (15)	0.41852 (15)	0.88474 (10)	0.0220 (3)	
H13A	−0.0127	0.5181	0.8984	0.026*	
H13B	−0.0188	0.3884	0.8658	0.026*	
C14	0.15997 (15)	0.42874 (14)	0.80402 (9)	0.0195 (3)	
H14A	0.1089	0.5005	0.7469	0.023*	
H14B	0.2219	0.4659	0.8210	0.023*	
C15	0.13013 (14)	0.29600 (13)	0.37536 (9)	0.0156 (2)	
C16	0.20321 (14)	0.27219 (13)	0.28691 (9)	0.0162 (2)	
H16	0.2144	0.3520	0.2402	0.019*	
C17	0.25856 (14)	0.13342 (14)	0.26797 (9)	0.0163 (2)	
C18	0.24168 (14)	0.01325 (14)	0.33586 (9)	0.0183 (3)	
H18	0.2780	−0.0812	0.3220	0.022*	
C19	0.17215 (14)	0.03538 (13)	0.42195 (9)	0.0175 (2)	
H19	0.1615	−0.0455	0.4678	0.021*	
C20	0.11576 (13)	0.17479 (13)	0.44478 (9)	0.0157 (2)	
C21	0.37176 (15)	0.21061 (15)	0.11990 (9)	0.0214 (3)	
H21A	0.4315	0.1707	0.0662	0.032*	
H21B	0.4280	0.2368	0.1512	0.032*	
H21C	0.2821	0.3000	0.0974	0.032*	
C22	0.05326 (13)	0.18669 (13)	0.53806 (9)	0.0158 (2)	
H22	0.0479	0.0999	0.5794	0.019*	
C23	−0.04492 (14)	0.27936 (13)	0.67398 (9)	0.0175 (2)	
H23A	0.0000	0.3175	0.7062	0.021*	
H23B	−0.0106	0.1710	0.7028	0.021*	
C24	−0.21159 (14)	0.35923 (14)	0.68823 (9)	0.0183 (3)	
H24A	−0.2481	0.4609	0.6459	0.022*	
H24B	−0.2553	0.3042	0.6709	0.022*	
C25	−0.22424 (19)	0.22319 (15)	0.85116 (10)	0.0287 (3)	
H25A	−0.1165	0.1614	0.8506	0.034*	
H25B	−0.2685	0.1715	0.8307	0.034*	
C26	−0.2839 (2)	0.24170 (16)	0.94997 (10)	0.0335 (4)	
H26A	−0.2591	0.1421	0.9939	0.040*	
H26B	−0.2373	0.2910	0.9708	0.040*	
C27	−0.47234 (17)	0.47365 (16)	0.89310 (10)	0.0264 (3)	
H27A	−0.4271	0.5223	0.9159	0.032*	
H27B	−0.5801	0.5354	0.8956	0.032*	
C28	−0.41669 (15)	0.46615 (16)	0.79223 (10)	0.0224 (3)	
H28A	−0.4690	0.4266	0.7673	0.027*	
H28B	−0.4378	0.5681	0.7529	0.027*	

### Atomic displacement parameters (Å^2^)

**Table:**

	*U*^11^	*U*^22^	*U*^33^	*U*^12^	*U*^13^	*U*^23^
Ni1	0.01396 (11)	0.01469 (11)	0.01148 (12)	−0.00886 (9)	0.00226 (8)	−0.00429 (8)
Ni2	0.01437 (11)	0.00926 (11)	0.01011 (12)	−0.00527 (8)	0.00115 (8)	−0.00286 (8)
O1	0.0239 (5)	0.0239 (5)	0.0250 (5)	−0.0154 (4)	0.0058 (4)	−0.0119 (4)
O2	0.0252 (5)	0.0203 (5)	0.0203 (5)	−0.0112 (4)	0.0053 (4)	−0.0080 (4)
O3	0.0262 (5)	0.0214 (5)	0.0158 (5)	−0.0079 (4)	0.0025 (4)	−0.0051 (4)
O4	0.0277 (5)	0.0132 (4)	0.0181 (5)	−0.0089 (4)	0.0029 (4)	−0.0051 (3)
O5	0.0245 (5)	0.0185 (4)	0.0187 (5)	−0.0098 (4)	0.0050 (4)	−0.0083 (4)
O6	0.0490 (7)	0.0321 (6)	0.0244 (6)	−0.0273 (5)	0.0166 (5)	−0.0102 (4)
N1	0.0167 (5)	0.0199 (5)	0.0139 (5)	−0.0098 (4)	0.0014 (4)	−0.0036 (4)
N2	0.0182 (5)	0.0162 (5)	0.0156 (5)	−0.0077 (4)	0.0016 (4)	−0.0039 (4)
N3	0.0180 (5)	0.0156 (5)	0.0141 (5)	−0.0078 (4)	−0.0004 (4)	−0.0030 (4)
N4	0.0209 (5)	0.0157 (5)	0.0137 (5)	−0.0085 (4)	0.0010 (4)	−0.0027 (4)
C1	0.0187 (6)	0.0156 (5)	0.0156 (6)	−0.0078 (5)	−0.0017 (5)	−0.0021 (4)
C2	0.0181 (6)	0.0158 (6)	0.0185 (6)	−0.0084 (5)	0.0004 (5)	−0.0027 (5)
C3	0.0188 (6)	0.0147 (6)	0.0146 (6)	−0.0052 (5)	−0.0012 (5)	−0.0013 (5)
C4	0.0239 (6)	0.0185 (6)	0.0196 (6)	−0.0102 (5)	−0.0026 (5)	−0.0052 (5)
C5	0.0201 (6)	0.0176 (6)	0.0182 (6)	−0.0104 (5)	−0.0029 (5)	−0.0017 (5)
C6	0.0181 (6)	0.0157 (5)	0.0144 (6)	−0.0079 (5)	−0.0025 (5)	−0.0011 (4)
C7	0.0235 (7)	0.0238 (7)	0.0240 (7)	−0.0115 (6)	0.0067 (6)	−0.0073 (5)
C8	0.0179 (6)	0.0176 (6)	0.0158 (6)	−0.0097 (5)	−0.0027 (5)	−0.0008 (4)
C9	0.0166 (6)	0.0217 (6)	0.0168 (6)	−0.0117 (5)	0.0018 (5)	−0.0046 (5)
C10	0.0169 (6)	0.0211 (6)	0.0171 (6)	−0.0102 (5)	0.0017 (5)	−0.0049 (5)
C11	0.0211 (6)	0.0183 (6)	0.0177 (6)	−0.0068 (5)	0.0010 (5)	−0.0040 (5)
C12	0.0266 (7)	0.0192 (6)	0.0170 (6)	−0.0101 (5)	0.0016 (5)	−0.0024 (5)
C13	0.0214 (6)	0.0215 (6)	0.0184 (7)	−0.0068 (5)	0.0015 (5)	−0.0044 (5)
C14	0.0213 (6)	0.0168 (6)	0.0185 (6)	−0.0081 (5)	0.0014 (5)	−0.0040 (5)
C15	0.0152 (6)	0.0143 (5)	0.0175 (6)	−0.0062 (5)	−0.0023 (5)	−0.0041 (4)
C16	0.0178 (6)	0.0148 (5)	0.0149 (6)	−0.0068 (5)	−0.0010 (5)	−0.0028 (4)
C17	0.0148 (6)	0.0187 (6)	0.0160 (6)	−0.0068 (5)	−0.0008 (5)	−0.0062 (5)
C18	0.0197 (6)	0.0149 (5)	0.0215 (7)	−0.0074 (5)	0.0001 (5)	−0.0072 (5)
C19	0.0188 (6)	0.0147 (5)	0.0201 (6)	−0.0088 (5)	−0.0015 (5)	−0.0029 (5)
C20	0.0158 (6)	0.0153 (5)	0.0170 (6)	−0.0075 (5)	−0.0020 (5)	−0.0036 (5)
C21	0.0230 (7)	0.0207 (6)	0.0177 (6)	−0.0090 (5)	0.0027 (5)	−0.0041 (5)
C22	0.0161 (6)	0.0148 (5)	0.0169 (6)	−0.0081 (5)	−0.0012 (5)	−0.0022 (4)
C23	0.0219 (6)	0.0156 (5)	0.0136 (6)	−0.0085 (5)	0.0006 (5)	−0.0021 (4)
C24	0.0206 (6)	0.0206 (6)	0.0158 (6)	−0.0116 (5)	0.0000 (5)	−0.0035 (5)
C25	0.0451 (9)	0.0177 (6)	0.0179 (7)	−0.0133 (6)	0.0060 (6)	−0.0025 (5)
C26	0.0531 (10)	0.0207 (7)	0.0174 (7)	−0.0136 (7)	0.0068 (7)	−0.0021 (5)
C27	0.0274 (7)	0.0302 (7)	0.0207 (7)	−0.0135 (6)	0.0075 (6)	−0.0090 (6)
C28	0.0206 (6)	0.0297 (7)	0.0189 (7)	−0.0126 (6)	0.0019 (5)	−0.0082 (5)

### Geometric parameters (Å, °)

**Table:**

Ni1—O1^i^	1.9001 (9)	C9—H9A	0.9900
Ni1—O1	1.9001 (9)	C9—H9B	0.9900
Ni1—N1^i^	2.0077 (10)	C10—H10A	0.9900
Ni1—N1	2.0077 (10)	C10—H10B	0.9900
Ni2—O4^ii^	1.8873 (9)	C11—C12	1.5136 (18)
Ni2—O4	1.8873 (9)	C11—H11A	0.9900
Ni2—N3^ii^	2.0105 (10)	C11—H11B	0.9900
Ni2—N3	2.0105 (10)	C12—H12A	0.9900
O1—C1	1.3052 (15)	C12—H12B	0.9900
O2—C3	1.3627 (16)	C13—C14	1.5180 (18)
O2—C7	1.4319 (16)	C13—H13A	0.9900
O3—C12	1.4237 (16)	C13—H13B	0.9900
O3—C13	1.4312 (16)	C14—H14A	0.9900
O4—C15	1.3048 (15)	C14—H14B	0.9900
O5—C17	1.3613 (16)	C15—C16	1.4140 (18)
O5—C21	1.4360 (15)	C15—C20	1.4225 (16)
O6—C26	1.419 (2)	C16—C17	1.3829 (17)
O6—C27	1.4244 (17)	C16—H16	0.9500
N1—C8	1.2928 (17)	C17—C18	1.4149 (17)
N1—C9	1.4786 (15)	C18—C19	1.3697 (18)
N2—C11	1.4633 (16)	C18—H18	0.9500
N2—C10	1.4650 (16)	C19—C20	1.4141 (17)
N2—C14	1.4700 (16)	C19—H19	0.9500
N3—C22	1.2974 (16)	C20—C22	1.4281 (18)
N3—C23	1.4740 (16)	C21—H21A	0.9800
N4—C24	1.4614 (17)	C21—H21B	0.9800
N4—C28	1.4631 (17)	C21—H21C	0.9800
N4—C25	1.4667 (16)	C22—H22	0.9500
C1—C2	1.4201 (18)	C23—C24	1.5235 (18)
C1—C6	1.4223 (17)	C23—H23A	0.9900
C2—C3	1.3830 (18)	C23—H23B	0.9900
C2—H2	0.9500	C24—H24A	0.9900
C3—C4	1.4129 (18)	C24—H24B	0.9900
C4—C5	1.3698 (19)	C25—C26	1.512 (2)
C4—H4	0.9500	C25—H25A	0.9900
C5—C6	1.4146 (17)	C25—H25B	0.9900
C5—H5	0.9500	C26—H26A	0.9900
C6—C8	1.4270 (18)	C26—H26B	0.9900
C7—H7A	0.9800	C27—C28	1.5109 (19)
C7—H7B	0.9800	C27—H27A	0.9900
C7—H7C	0.9800	C27—H27B	0.9900
C8—H8	0.9500	C28—H28A	0.9900
C9—C10	1.5197 (18)	C28—H28B	0.9900
			
O1^i^—Ni1—O1	180.00 (5)	O3—C12—H12B	109.3
O1^i^—Ni1—N1^i^	91.36 (4)	C11—C12—H12B	109.3
O1—Ni1—N1^i^	88.64 (4)	H12A—C12—H12B	108.0
O1^i^—Ni1—N1	88.64 (4)	O3—C13—C14	111.45 (11)
O1—Ni1—N1	91.36 (4)	O3—C13—H13A	109.3
N1^i^—Ni1—N1	180.000 (1)	C14—C13—H13A	109.3
O4^ii^—Ni2—O4	180.000 (1)	O3—C13—H13B	109.3
O4^ii^—Ni2—N3^ii^	91.43 (4)	C14—C13—H13B	109.3
O4—Ni2—N3^ii^	88.57 (4)	H13A—C13—H13B	108.0
O4^ii^—Ni2—N3	88.57 (4)	N2—C14—C13	110.41 (10)
O4—Ni2—N3	91.43 (4)	N2—C14—H14A	109.6
N3^ii^—Ni2—N3	180.00 (8)	C13—C14—H14A	109.6
C1—O1—Ni1	129.48 (8)	N2—C14—H14B	109.6
C3—O2—C7	117.40 (10)	C13—C14—H14B	109.6
C12—O3—C13	109.77 (10)	H14A—C14—H14B	108.1
C15—O4—Ni2	131.19 (8)	O4—C15—C16	117.97 (11)
C17—O5—C21	117.05 (10)	O4—C15—C20	123.00 (12)
C26—O6—C27	108.78 (11)	C16—C15—C20	119.02 (11)
C8—N1—C9	115.10 (10)	C17—C16—C15	120.42 (11)
C8—N1—Ni1	123.43 (9)	C17—C16—H16	119.8
C9—N1—Ni1	121.41 (8)	C15—C16—H16	119.8
C11—N2—C10	110.51 (10)	O5—C17—C16	124.33 (11)
C11—N2—C14	108.13 (10)	O5—C17—C18	114.78 (11)
C10—N2—C14	112.35 (10)	C16—C17—C18	120.89 (12)
C22—N3—C23	115.73 (10)	C19—C18—C17	118.91 (11)
C22—N3—Ni2	124.12 (9)	C19—C18—H18	120.5
C23—N3—Ni2	120.03 (8)	C17—C18—H18	120.5
C24—N4—C28	109.14 (10)	C18—C19—C20	122.07 (11)
C24—N4—C25	111.97 (10)	C18—C19—H19	119.0
C28—N4—C25	109.90 (11)	C20—C19—H19	119.0
O1—C1—C2	118.41 (11)	C19—C20—C15	118.67 (12)
O1—C1—C6	123.26 (12)	C19—C20—C22	118.84 (11)
C2—C1—C6	118.33 (12)	C15—C20—C22	122.41 (11)
C3—C2—C1	120.54 (12)	O5—C21—H21A	109.5
C3—C2—H2	119.7	O5—C21—H21B	109.5
C1—C2—H2	119.7	H21A—C21—H21B	109.5
O2—C3—C2	124.17 (11)	O5—C21—H21C	109.5
O2—C3—C4	114.60 (12)	H21A—C21—H21C	109.5
C2—C3—C4	121.22 (12)	H21B—C21—H21C	109.5
C5—C4—C3	118.52 (12)	N3—C22—C20	127.44 (11)
C5—C4—H4	120.7	N3—C22—H22	116.3
C3—C4—H4	120.7	C20—C22—H22	116.3
C4—C5—C6	122.20 (12)	N3—C23—C24	111.20 (10)
C4—C5—H5	118.9	N3—C23—H23A	109.4
C6—C5—H5	118.9	C24—C23—H23A	109.4
C5—C6—C1	119.11 (12)	N3—C23—H23B	109.4
C5—C6—C8	118.53 (11)	C24—C23—H23B	109.4
C1—C6—C8	122.35 (12)	H23A—C23—H23B	108.0
O2—C7—H7A	109.5	N4—C24—C23	111.74 (11)
O2—C7—H7B	109.5	N4—C24—H24A	109.3
H7A—C7—H7B	109.5	C23—C24—H24A	109.3
O2—C7—H7C	109.5	N4—C24—H24B	109.3
H7A—C7—H7C	109.5	C23—C24—H24B	109.3
H7B—C7—H7C	109.5	H24A—C24—H24B	107.9
N1—C8—C6	127.62 (11)	N4—C25—C26	109.78 (11)
N1—C8—H8	116.2	N4—C25—H25A	109.7
C6—C8—H8	116.2	C26—C25—H25A	109.7
N1—C9—C10	110.54 (10)	N4—C25—H25B	109.7
N1—C9—H9A	109.5	C26—C25—H25B	109.7
C10—C9—H9A	109.5	H25A—C25—H25B	108.2
N1—C9—H9B	109.5	O6—C26—C25	111.08 (14)
C10—C9—H9B	109.5	O6—C26—H26A	109.4
H9A—C9—H9B	108.1	C25—C26—H26A	109.4
N2—C10—C9	111.81 (10)	O6—C26—H26B	109.4
N2—C10—H10A	109.3	C25—C26—H26B	109.4
C9—C10—H10A	109.3	H26A—C26—H26B	108.0
N2—C10—H10B	109.3	O6—C27—C28	111.46 (12)
C9—C10—H10B	109.3	O6—C27—H27A	109.3
H10A—C10—H10B	107.9	C28—C27—H27A	109.3
N2—C11—C12	109.55 (11)	O6—C27—H27B	109.3
N2—C11—H11A	109.8	C28—C27—H27B	109.3
C12—C11—H11A	109.8	H27A—C27—H27B	108.0
N2—C11—H11B	109.8	N4—C28—C27	111.16 (12)
C12—C11—H11B	109.8	N4—C28—H28A	109.4
H11A—C11—H11B	108.2	C27—C28—H28A	109.4
O3—C12—C11	111.61 (10)	N4—C28—H28B	109.4
O3—C12—H12A	109.3	C27—C28—H28B	109.4
C11—C12—H12A	109.3	H28A—C28—H28B	108.0
			
N1^i^—Ni1—O1—C1	−162.51 (11)	C13—O3—C12—C11	58.29 (14)
N1—Ni1—O1—C1	17.49 (11)	N2—C11—C12—O3	−60.30 (15)
N3^ii^—Ni2—O4—C15	173.30 (12)	C12—O3—C13—C14	−56.79 (14)
N3—Ni2—O4—C15	−6.70 (12)	C11—N2—C14—C13	−57.87 (14)
O1^i^—Ni1—N1—C8	166.44 (11)	C10—N2—C14—C13	179.90 (11)
O1—Ni1—N1—C8	−13.56 (11)	O3—C13—C14—N2	57.68 (15)
O1^i^—Ni1—N1—C9	−10.58 (9)	Ni2—O4—C15—C16	−170.87 (9)
O1—Ni1—N1—C9	169.42 (9)	Ni2—O4—C15—C20	8.59 (19)
O4^ii^—Ni2—N3—C22	−177.97 (11)	O4—C15—C16—C17	−179.89 (11)
O4—Ni2—N3—C22	2.03 (11)	C20—C15—C16—C17	0.63 (18)
O4^ii^—Ni2—N3—C23	−2.22 (9)	C21—O5—C17—C16	8.00 (18)
O4—Ni2—N3—C23	177.78 (9)	C21—O5—C17—C18	−171.33 (11)
Ni1—O1—C1—C2	166.26 (9)	C15—C16—C17—O5	−178.45 (12)
Ni1—O1—C1—C6	−12.93 (19)	C15—C16—C17—C18	0.85 (19)
O1—C1—C2—C3	−177.25 (12)	O5—C17—C18—C19	177.94 (12)
C6—C1—C2—C3	1.99 (18)	C16—C17—C18—C19	−1.43 (19)
C7—O2—C3—C2	−6.01 (18)	C17—C18—C19—C20	0.51 (19)
C7—O2—C3—C4	174.14 (11)	C18—C19—C20—C15	0.94 (19)
C1—C2—C3—O2	176.76 (11)	C18—C19—C20—C22	−175.93 (12)
C1—C2—C3—C4	−3.39 (19)	O4—C15—C20—C19	179.04 (12)
O2—C3—C4—C5	−177.91 (11)	C16—C15—C20—C19	−1.50 (18)
C2—C3—C4—C5	2.23 (19)	O4—C15—C20—C22	−4.20 (19)
C3—C4—C5—C6	0.3 (2)	C16—C15—C20—C22	175.25 (12)
C4—C5—C6—C1	−1.59 (19)	C23—N3—C22—C20	−175.40 (12)
C4—C5—C6—C8	179.30 (12)	Ni2—N3—C22—C20	0.52 (19)
O1—C1—C6—C5	179.65 (12)	C19—C20—C22—N3	176.48 (12)
C2—C1—C6—C5	0.45 (18)	C15—C20—C22—N3	−0.3 (2)
O1—C1—C6—C8	−1.3 (2)	C22—N3—C23—C24	−108.75 (12)
C2—C1—C6—C8	179.52 (11)	Ni2—N3—C23—C24	75.15 (12)
C9—N1—C8—C6	−176.67 (12)	C28—N4—C24—C23	171.84 (10)
Ni1—N1—C8—C6	6.13 (19)	C25—N4—C24—C23	−66.25 (14)
C5—C6—C8—N1	−176.71 (12)	N3—C23—C24—N4	−165.34 (10)
C1—C6—C8—N1	4.2 (2)	C24—N4—C25—C26	−176.26 (12)
C8—N1—C9—C10	101.55 (13)	C28—N4—C25—C26	−54.79 (17)
Ni1—N1—C9—C10	−81.20 (11)	C27—O6—C26—C25	−61.61 (15)
C11—N2—C10—C9	167.06 (10)	N4—C25—C26—O6	60.01 (17)
C14—N2—C10—C9	−72.06 (13)	C26—O6—C27—C28	59.59 (16)
N1—C9—C10—N2	−175.82 (10)	C24—N4—C28—C27	176.61 (11)
C10—N2—C11—C12	−177.93 (10)	C25—N4—C28—C27	53.46 (15)
C14—N2—C11—C12	58.71 (13)	O6—C27—C28—N4	−56.47 (16)

Symmetry codes: (i) −*x*+1, −*y*+1, −*z*+1; (ii) −*x*, −*y*+1, −*z*+1.
